# Anticipation of difficult tasks: neural correlates of negative emotions and emotion regulation

**DOI:** 10.1186/s12993-019-0155-1

**Published:** 2019-03-18

**Authors:** Elise Klein, Silke M. Bieck, Johannes Bloechle, Stefan Huber, Julia Bahnmueller, Klaus Willmes, Korbinian Moeller

**Affiliations:** 1 Leibniz-Institut für Wissensmedien, Tübingen, Germany; 2 LEAD Graduate School and Research Network, University of Tuebingen, Tübingen, Germany; 3 Department of Psychiatry and Psychotherapy, University of Tuebingen, Tübingen, Germany; 4 Department of Psychology, University of Tuebingen, Tübingen, Germany; 5 Department of Neurology, University Hospital, RWTH Aachen University, Aachen, Germany

**Keywords:** Task difficulty, Difficult math, Cognition and emotion, Emotion regulation, fMRI

## Abstract

**Background:**

Difficult cognitive tasks are often associated with negative feelings. This can be already the case for the mere anticipation of having to do a difficult task. For the case of difficult math tasks, it was recently suggested that such a negative emotional response may be exclusive to highly math-anxious individuals. However, it is also conceivable that negative emotional responses simply reflect that math is perceived as difficult. Here we investigated whether non-math-anxious individuals also experience negative emotional responses when anticipating to do difficult math tasks.

**Methods:**

We compared brain activation following the presentation of a numerical cue indicating either difficult or easy upcoming proportion magnitude comparison tasks.

**Results:**

Comparable to previous results for highly math-anxious individuals we observed a network associated with negative emotions to be activated in non-math-anxious individuals when facing cues indicating a difficult upcoming task. Importantly, however, math anxiety scores did not predict the neural response. Furthermore, we observed activation in areas associated with processes of cognitive control areas such as anterior cingulate cortex, which were suggested to play a key role in emotion regulation.

**Conclusion:**

Activation in the emotion processing network was observed when anticipating an upcoming difficult (math) task. However, this activation was not predicted by individual’ degree of math anxiety. Therefore, we suggest that negative emotional responses to difficult math tasks might be a rather common reaction not specific to math-anxious individuals. Whether or not this initial negative response impairs math performance, however, might depend on the ability to regulate those emotions effectively.

**Electronic supplementary material:**

The online version of this article (10.1186/s12993-019-0155-1) contains supplementary material, which is available to authorized users.

## Introduction

Research repeatedly showed that doing difficult tasks is often associated with negative feelings such as high arousal, stress or anxiety. In particular, these negative feelings seem to be more pronounced for difficult as compared to more easy tasks (see [1] for a review) and can negatively affect cognitive performance on a variety of tasks (e.g., reading, attention, etc. [2–4]). In this context, difficulty is typically characterized by the demand for attentional, cognitive, etc. resources needed to master the task at hand. This way, a difficult (i.e., more demanding) task in turn leads to compromised performance because arousal, stress and anxiety consume cognitive resources (e.g., [5]). This may even end in a vicious circle where the individual perceives demanding tasks as threatening, which then leads to more anxiety. Anxiety is typically defined as a negative emotional state that occurs in situations in which the level of perceived demands to the individual is experienced as outweighing her/his resources to complete the task at hand [6].

The relationship between task difficulty and performance is reflected by cortical activation during task performance. One assumption is that anxious individuals worry more about a demanding and potentially threatening task and how to cope with it. As a result, these anxious participants try to employ strategies to reduce effects of anxiety to master the task, which is reflected by enhanced activation in amygdala and reduced recruitment of areas associated with cognitive control and inhibition such as dorsolateral prefrontal cortex (DLPFC; see [7], for a review). In contrast, non-anxious participants might also feel anxious about a difficult upcoming task but then, however, successfully employ processes of emotion-regulation (e.g., [8]) to reappraise negative feelings in unemotional terms, which may be reflected by increased activation in these areas associated with cognitive control.

Interestingly, negative feelings are also often reported in the context of mathematical tasks (e.g., [7–9]). For instance, dealing with numbers was shown to induce intense negative emotions and stress so reliably that mental arithmetic is one of the most commonly used tasks to induce stress in laboratory settings (e.g., [10–13]). Increased stress levels may, for instance, be reflected by increases in heart rate and blood pressure (e.g., [14, 15]). It is possible that these strong negative reactions to mathematical tasks reflect a specific anxiety associated with numerical tasks (i.e., math anxiety, e.g., [9]). It is, however, also conceivable that the negative reactions simply reflect the fact that math is perceived as difficult. Interestingly, more difficult numerical tasks seem to elicit stronger negative emotions and physiological responses than easier numerical tasks (e.g., [16, 17]; see also [8] for neural correlates). Therefore, probably both, difficult tasks as well as numerical tasks are associated with negative feelings in general, so that the strongest emotional and physiological response to math should be observed when participants have to solve difficult mathematical tasks [8] in particular.

As mentioned above, it is possible that strong negative reactions to mathematical tasks simply reflect that math is perceived difficult. Nevertheless, there is a rich body of literature on math anxiety, showing that if such negative emotional responses to math or the anticipation of having to do math cannot be regulated or compensated, performance in numerical tasks is significantly reduced in a wide variety of everyday life and academic situations. In this case, affected individuals are classified as math-anxious (e.g., [18, 19]; for a review see [20]). Math-anxious individuals were observed repeatedly to perform poorly in tasks which involve numerical information, while their performance in other general reasoning tasks is not affected and typical (e.g., [21, 22]).

In order to deepen our understanding of underlying mechanisms leading to decreased numerical information processing in the context of difficult math tasks, research on the neuro-cognitive underpinnings of negative emotional reactions while doing math is highly relevant. However, it was suggested that a negative emotional response to mathematic is only observed in high math-anxious participants [8, 9]. Moreover, because emotional responses to math are strongest when participants have to solve difficult mathematical tasks, Lyons and Beilock [8, 9] evaluated neural activation patterns in response to difficult tasks from participants performing both, difficult and easy numerical and non-numerical tasks. Importantly, the authors evaluated neural activation during the actual completion of the task [8] as well as during the mere anticipation of having to do the respective numerical task, this means, following presentation of a cue indicating the nature of the upcoming task (i.e., difficult vs. easy [8, 9]). The authors observed that already after the presentation of a cue indicating a difficult upcoming numerical task and thus before the respective task has to be performed, brain regions associated with the processing of negative emotions [8] and even pain [9] were activated. The network for negative emotions comprised bilateral hippocampus and (pre)frontal areas [8], but not the amygdala, which is typically activated in emotion processing [23], whereas the pain network included the insula and middle cingulate cortex [8].

However, Lyons and Beilock [8, 9] reported that math-anxious participants, who showed typical and thus unimpaired performance in math tasks, activated not only neural networks associated with the processing of negative emotions and pain, but also a network associated with cognitive control processes. This cognitive control network was argued to be involved in regulating negative feelings of fear, despair or pain by reappraisal and involved dorsolateral prefrontal cortex (DLPFC) and anterior cingulate cortices (ACC). Importantly, areas forming this cognitive control network also seem to play a key role in emotion regulation more generally (e.g., [24]; for a review see [25]).

Interestingly, neural networks for processing negative emotions and pain as well as for evincing cognitive control were shown to be activated already when participants anticipated the upcoming numerical task. This indicates that emotional effects associated with math can already be observed when investigating the time interval between cue presentation and the beginning of the actual task [8, 9] and these emotional effects are thus not confounded by neural activation associated with actually performing the task. In case emotion regulation is successful, the task at hand may then be performed with all available cognitive resources [21] so that performance should not be impaired [8].

However, it is important to note that the latter assumptions of Lyons and Beilock (i.e., no impairment of performance with successful emotion regulation) are based on the comparison of highly math-anxious with low math-anxious participants (i.e., participants scoring in the upper vs. in the lower quintile in a math anxiety screening test [8]) and on a comparison within highly math-anxious participants [9]. According to the authors, the network associated with processing negative emotions should only be observed in highly math-anxious participants because low math anxious persons “[…] do not have a negative emotional response in anticipation of math that requires reinterpreting” ([8], p. 2108). If this assumption was true, one should neither be able to observe activation within this network for processing negative emotions in non-math-anxious participants when anticipating a difficult upcoming numerical task, nor should non-math-anxious participants express negative feelings when asked after task completion (e.g., in a questionnaire). Additionally, they should not show activation in the cognitive control network subserving the regulation of emotions.

However, there is evidence showing that (i) negative emotional responses in anticipation of a difficult task seem to be more like a general tendency rather than an exception (cf. [26] for a review) and (ii) that these negative emotions can be successfully regulated using reappraisal [27] and mechanisms of cognitive control [24, 28]. According to the process model of emotion regulation, reappraisal is an early emotional control process and provides one of the most effective means to diminish the negative emotions associated with an aversive event [24, 27, 29]. When the regulation of the initial emotional response is successful, task performance will be better, as demonstrated by Lyons and Beilock [8, 9] for the case of highly math-anxious participants. Therefore, we hypothesize that non-math-anxious participants should not necessarily be characterized by the absence of activation in the network associated with processing negative emotions. Instead non-math-anxious participants should be characterized by the presence of (sufficient) activation in networks subserving cognitive control/emotion regulation during number processing—an interplay which was shown by Lyons and Beilock [9] for highly math-anxious participants with normal math performance.

### The present study

**Fig. 1:**
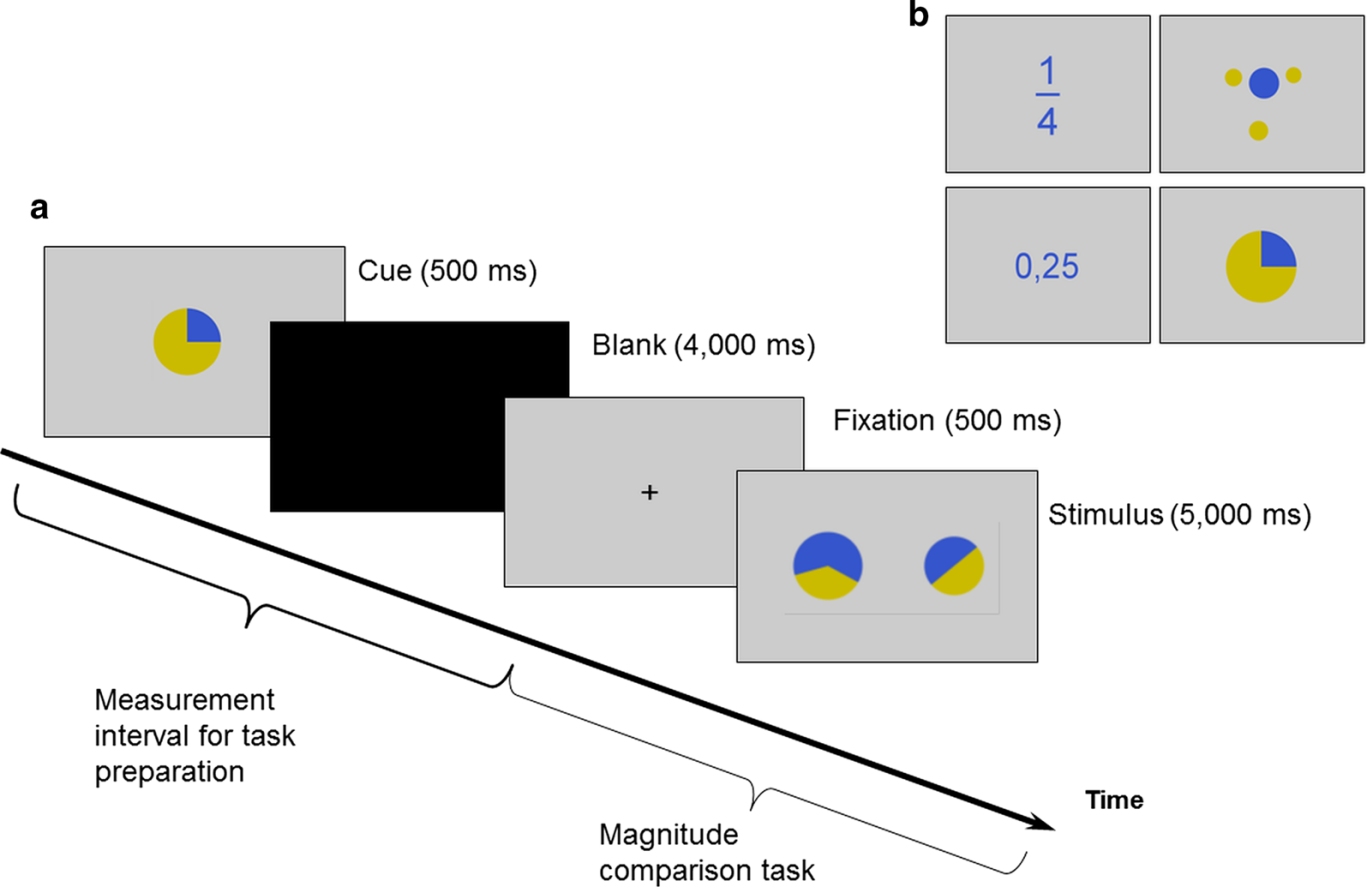
Experimental design. **a** Illustration of the experimental procedure at the beginning of each block (i.e., one out of five trials). **b** Presented cues in all notation formats

In this study, we aimed at investigating neural activation of non-math anxious participants during the anticipation of difficult and easy numerical tasks. We used a numerical task because math is typically perceived as difficult and demanding. In particular, we employed a comparison task on relative magnitudes (i.e., fractions and proportions) and investigated neural activation in response to cues indicating these upcoming tasks. We used magnitude comparison of proportions, because fractions and proportions are difficult enough to elicit emotional responses in non-math-anxious participants. At the same time, proportions are also well suited for manipulating task difficulty. To avoid that observed effects are driven by notation-specific processes, we used both symbolic and non-symbolic proportions. This is important because we wanted to evaluate rather general cognitive processing mechanisms. Therefore, we did not expect to observe specific IPS activation, because the IPS is generally assumed to subserve the processing of number magnitude in a notation independent manner [30–33]. As it was found that magnitude comparison of decimals is easier than magnitude comparison of fractions [34], we used fraction comparison as the more difficult and decimal comparison as the easier condition in symbolic notation. For non-symbolic notation, we used proportions visualized by dot patterns (i.e., the relation of blue to yellow dots, see also Fig. 1) as the more difficult and pie charts as the easier condition according to behavioral pilot data.

Moreover, to complement neural activation data with a subjective measure of emotional responses and to determine whether these responses were actually negative or positive, participants also answered a stress appraisal questionnaire after completing the tasks. We hypothesized that negative emotional responses to the anticipation of doing difficult math are present in all individuals irrespective of their math-anxiety level. In this case, it would be less important how strong this emotional response is rather than how well it can be regulated.

Thus, we hypothesized that the more difficult tasks should elicit stronger negative emotional responses in our non-math-anxious participants both subjectively (as shown in a questionnaire) and objectively (as shown by neural activations during anticipation) independent of presentation notation. Since we evaluated non-math-anxious participants, we also expected concurrent activation in a network associated with cognitive control reflecting emotion regulation. In order to determine the influence of math anxiety values subjectively assessed with the Abbreviated Math Anxiety Scale (AMAS; [35]) on activation patterns, the activation predicted by math anxiety values was modelled separately.

In addition to this hypothesis on notation-independent processes of emotion regulation, we also expected specific differences in activation patterns for symbolic digital and non-symbolic cues. In particular, we expected to find activation in areas associated with the identification of Arabic digits such as the visual number form (VNF) following symbolic cues (i.e., fractions, decimals). As the VNF is supposed to be automatically involved in the processing of visual numerals [36, 37], whenever one visually perceives symbolic numerical stimuli.

## Methods and materials

### Participants

25 right-handed adult volunteers (13 female, mean age = 23.2 years; SD = 2.99 years) participated in the study. Written informed consent was obtained from all participants. The experiment was approved by the local Ethics Committee of the Medical Faculty of the University of Tuebingen (274/2013BO2). All participants reported normal or corrected to normal vision and no previous history of neurological or psychiatric disorders. In particular, neurological and psychiatric disorders were assessed using both, a detailed self-assessment questionnaire and an diagnostic questionnaire, which was completed by a specifically trained and certified MR investigator to rule out anxiety disorders according to DSM-5 [38]. Moreover, participants were not math anxious according to the Abbreviated Math Anxiety Scale (AMAS; [35]). Only participants who were not taking any medications other than oral contraceptives were included in the study.

### Stimuli and design

In the magnitude comparison task, participants had to decide which of two presented proportions was the larger one. We used four different presentation notations of proportions: fractions, decimals, pie charts, and dot patterns (see Fig. 1a for an example). Each block of magnitude comparison tasks was preceded by a cue indicating the respective proportion notation to be expected on the next five trials. The cue always was the proportion 1/4 shown in the different notations in the center of the screen against a grey background (see Fig. 1b). A total of 24 items entered the cues analysis. Importantly, we evaluated only the neural response following cue presentation, but not the activation during the actual magnitude comparison.

For the magnitude comparison tasks, we constructed 30 items for each of the four presentation notations. Proportions were presented in pairs with the magnitude of the first proportion ranging from 0.13 to 0.86 and the second proportion ranging from 0.22 to 0.89. Absolute distances between proportions ranged from 0.02 to 0.22.

Before and after completing the proportion comparison task within the scanner, participants were asked to rate their anticipated feelings regarding the four presentation notations using an adapted version of the Stress Appraisal Measure [39, 40] provided as paper and pencil questionnaire. In this Stress Appraisal Measure, six items (three items each) assessed challenge (positive emotional valence) and threat (negative emotional valence) participants experienced related to the task (e.g., challenge, positive: “I think I can master these tasks”; threat, negative: “I am afraid of not being able to solve the tasks”). The idea is that individuals assess whether they can master a difficult task by weighing the perceived task demands (e.g., task effort) against their perceived resources (e.g., skills; [41, 42]). A task is considered as a threat when their task demands seem to outweigh their resources or as a challenge when their resources seem to match or exceed task demands [42, 43].

### Procedure

Before entering the scanner, participants completed the Stress Appraisal Measure. Then participants were put into the scanner and stimuli were projected on a screen above their head. Participants viewed the stimuli through a mirror mounted on the head coil of the scanner. Fractions as well as decimals were presented in blue (RGB-values: 53, 85, 204; font type: Arial; font size: 80) against a grey background (RGB-values: 204, 204, 204). Pie charts were drawn by dividing circles into two pie segments, one depicted in blue (same blue as for fractions) and the other in yellow (RGB-values: 203, 187, 0; see Fig. 1b) against the same grey background color. Dot patterns were colored according to the fractions they denoted using the same colors as for the pie charts.

Head movements were prevented by using foam pads. To familiarize participants with the task, all volunteers were given the opportunity to practice on several items of each condition preceded by the respective cues before starting the actual experiment. None of these practice items was repeated during the critical measurement.

At the beginning of each block a cue was presented for 500 ms that indicated the respective proportion notation to be expected on the next five trials. Subsequently, a black screen was presented for 4000 ms. To investigate processes associated with handling inherent numerical features of and affective associations with the numerical cues, we chose a design, in which the presentation of a cue was followed by a long time interval (4000 ms) with no further visual input until the cued task was actually presented. As pointed out by Brass and von Cramon [44], it might be difficult to otherwise isolate task preparation from task execution using neuroimaging methods (see also [8, 9]). While the time needed to prepare for a task may be very short, the hemodynamic response is comparably slow (i.e., peaking at about 6 s post stimulus, cf. [45]). This can possibly lead to an overlap of the hemodynamic responses for the cue and target period.

After 4000 ms, comparison trials were presented starting with a black fixation cross on a grey background for 500 ms, followed by the presentation of a proportion comparison stimulus for up to 5000 ms (see Fig. 1a for an illustration of the procedure at the beginning of a block). Participants had to respond within this time frame by pressing one of two MRI compatible response buttons with either their left (indicating left proportion larger) or right thumb (indicating right proportion larger). Proportion comparison items were presented in six blocks of five items each. After one block was completed, the next block was introduced by the next cue. We focused our analyses of neural activation observed during cue presentation and the following 4000 ms of a blank screen. Activation during the actual comparison of proportions was not considered in the present study.

### (f)MRI acquisition

MRI data were acquired using a 3T Siemens Magnetom TrioTim MRI system (Siemens AG, Erlangen, Germany). A high resolution T1—weighted anatomical scan (TR = 2300 s, matrix = 256 × 256, 176 slices, voxel size = 1.0 × 1.0 × 1.0 mm^3^; FOV = 256 mm^2^, TE = 2.92 ms; flip angle = 8°) was collected at the end of the experimental session. All functional measurements covered the whole brain using standard echo-planar imaging sequences (TR = 2400 ms; TE = 30 ms; flip angle = 80°; FOV = 220 mm^2^, 88 × 88 matrix; 42 slices, voxel size = 2.5 × 2.5 × 3.0 mm^3^, gap = 10%). fMRI data was acquired in a single run. Total scanning time was approximately 20 min. We included pauses between blocks in which a black screen was presented for 6000 ms.

## Analysis

### Behavioral analysis

Because the primary focus of the current study was on the neural correlates of processing numerical cues for task preparation activation, we only report the analysis of behavioral data with the view of a manipulation check for our 2 × 2 manipulation of task difficulty and notation.

We inspected response times (RT) and error rates (ER) to examine whether the difficulty of the presentation notations differed. Initial inspection of the behavioral data indicated that the distribution of response times was strongly skewed to the right, in particular for decimals (skewness: 2.608, SD = 0.456) and pies (skewness: 2.426, SD = 0.456). Therefore, we applied the inverse transformation converting response times into speed with measurement unit 1/s to approach normal distribution [46]. This way larger values indicate faster speed, while smaller values indicate slower responses.

Speed and error rates were analyzed running (generalized) linear mixed models [(G)LME] to include random effects for both participants and items (e.g., [47]). In the GLME (for ER), we used the logit as the link function and assumed a binomial error distribution. We included the fixed effect of presentation notation (fractions, decimals, pie charts, and dot patterns) and random intercepts for participants as well as items (crossed random effects), and a random slope for presentation notation in the LME as well as the GLME.

In the analysis of speed, we considered correctly solved trials only. Moreover, we removed trials with absolute z-scaled residuals of the full model larger than 3. In total, the analysis of speed was based on 82.6% of all trials. Data from the appraisal questionnaire completed after the experiment on negative (‘threat’) as well as positive (“challenge”) emotions towards the four conditions were analyzed each by a 2 × 2 repeated measures ANOVA discerning the two factors task difficulty (difficult vs. easy) and notation (symbolic vs. non-symbolic).

Statistical analyses were run using R [48] and the R packages lme4 [49] and afex for executing the (G)LME [50]. *p*-values for fixed effects of LME were calculated running F-tests using the Kenward–Roger approximation for degrees of freedom (e.g., [51]) and for GLME, we carried out likelihood ratio tests (LRT). Post-hoc tests were run relying on the R package lsmeans [52] and the Tukey HSD (honestly significant difference) method was used to adjust *p*-values for multiple comparisons. Plots were drawn using the R packages ggplot2 [53] and cowplot [54].

### Imaging analysis

Imaging data analysis was performed using SPM12 (http://www.fil.ion.ucl.ac.uk/spm). Images were slice-time corrected, motion corrected, and realigned to each participant’s mean image. The mean image was co-registered with the anatomical whole-brain volume. Imaging data was then normalized into standard stereotaxic MNI space (Montreal Neurological Institute, McGill University, Montreal, Canada). Images were resampled every 2.5 mm using 4th degree spline interpolation to obtain isovoxels and then smoothed with a 6 mm full-width at half-maximum (FWHM) Gaussian kernel to accommodate inter-subject variation in brain anatomy and to increase signal-to-noise ratio in the images. Data were high-pass filtered (128 s) to remove low-frequency noise components and corrected for autocorrelation assuming an AR(1) process.

In the first-level analysis, the onsets of the cues for the four presentation formats (i.e., fractions, decimals, pie charts, dot patterns) were entered as separate conditions in the GLM. Importantly, the neural response associated with the critical items was evaluated from the beginning of each cue presentation until the start of the fixation cross preceding the magnitude comparison task (duration of 4500 ms). Thus, activation during the actual comparison of proportions was not considered in the present analysis. Movement parameters estimated at the realignment stage of preprocessing were included as covariates of no interest. Motion parameters did not exceed 2.5 mm translation in total (i.e., they did not exceed voxel size) and a head rotation of 1.5° in pitch, roll, and yaw in total. Therefore, none of the participants had to be excluded from the analyses because of head movements. Brain activity was convolved over all experimental trials with the canonical hemodynamic response function (HRF) as implemented in SPM12 and its time and dispersion derivatives.

These contrast images then entered the second-level random-effects group analysis. The second-level analysis was realised using a flexible factorial design for repeated measures with difficulty (easy/difficult) and notation (symbolic/non-symbolic) as within-subject factors as well as math anxiety (AMAS score) as covariate. We evaluated both, main effects of difficulty and notation as well as the interaction between the two factors. Additionally, we evaluated the fMRI signal explained by low or high values of the covariate math anxiety.

The SPM Anatomy Toolbox [55], available for all published cytoarchitectonic maps (http://www.fz-juelich.de/ime/spm_anatomy_toolbox), was used for anatomical localization of effects where applicable. In areas not yet implemented, the anatomical automatic labelling tool (AAL) in SPM12 (http://www.cyceron.fr/web/aalanatomical_automatic_labeling.html) was applied. Activations were thresholded at an uncorrected *p*-value of < 0.001 at the voxel level with a cluster size of *k* = 10 voxels and were reported when they remained significant following family-wise error correction (FWE) at the cluster-level with *p*_cluster-corr_ < 0.05.

## Results

### Behavioral results

**Fig. 2:**
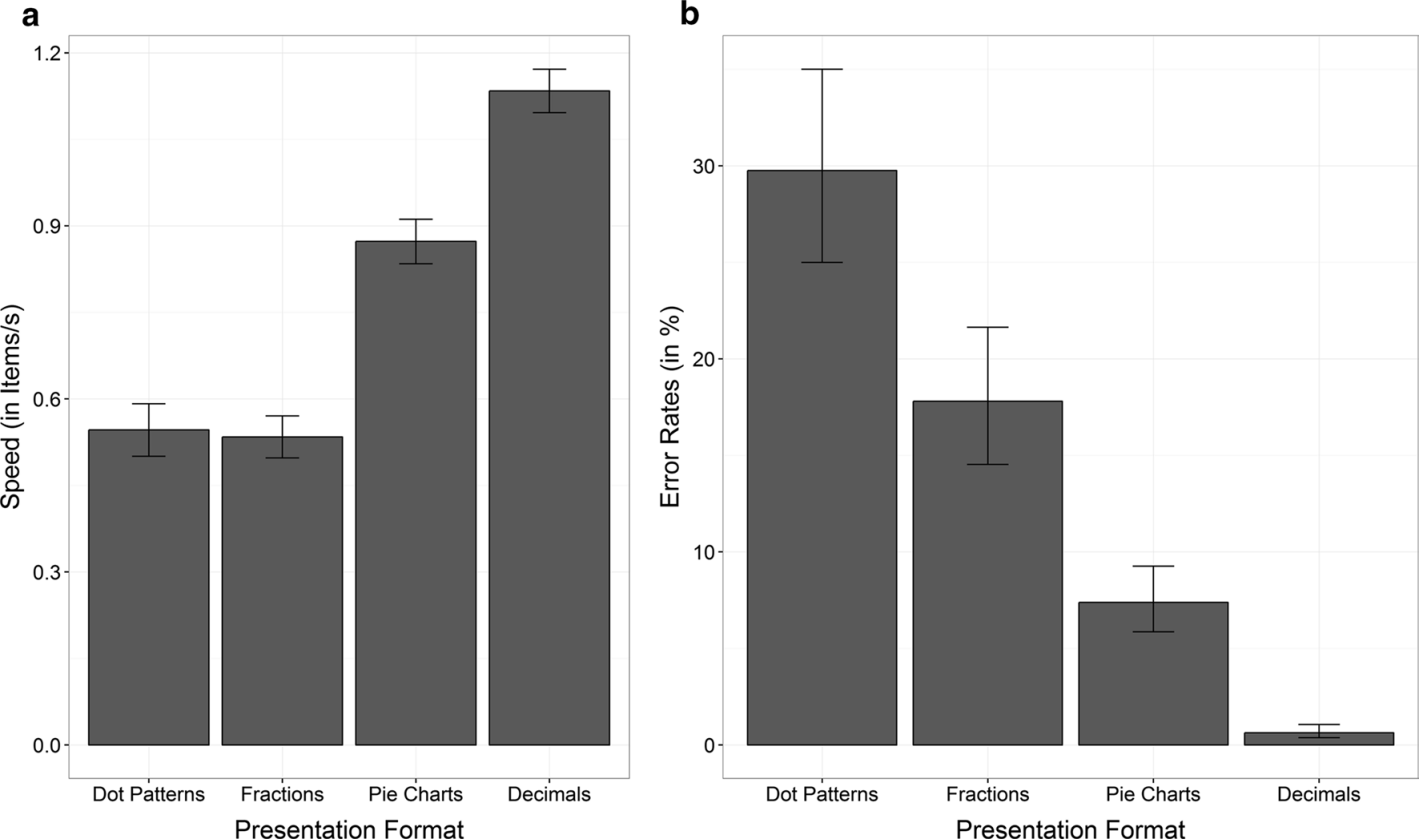
Behavioral data as manipulation check. **a** Mean speed and **b** error rates in the four conditions (dot patterns, pie charts, fractions, and decimals). Error bars indicate one standard error of the mean

The (ordinal) interaction between notation and difficulty was significant for speed, *F*(1, 24.45) = 78.96, *p* < 0.001 (see Fig. 2a) indicating that the effect of the factor difficulty was more pronounced for symbolic as compared to non-symbolic notation.

We further inspected the interaction by running pairwise post hoc comparisons among conditions. We found that speed differed significantly between these different presentation formats [dot patterns vs. pie charts: *t*(28.70) = 8.65, *p* < 0.001; dot patterns vs. decimals: *t*(24.37) = 12.64, *p* < 0.001; fractions vs. pie charts: *t*(26.33) = 10.04, *p* < 0.001; fractions vs. decimals: *t*(29.71) = 18.08, *p* < 0.001; pie charts vs. decimals: *t*(28.53) = 9.48, *p* < 0.001], except for dot patterns and fractions [*t*(32.28) = 0.39, *p* = 0.980]. Thus, decimals were compared fastest (*M* = 1.34 item/s, *SE* = 0.04 items/s), followed by pie charts (*M* = 0.87 item/s, *SE* = 0.04 items/s), whereas fractions (*M* = 0.54 item/s, *SE* = 0.04 items/s) and dot patterns (*M* = 0.55 item/s, *SE* = 0.05 items/s) were compared about equally fast. Moreover, main effects of notation, *F*(1, 27.75) = 24.93, *p* < 0.001, and difficulty, *F*(1, 24.24) = 208.88, *p* < 0.001, were significant. However, the main effect of notation should not be interpreted, because the (simple) main effect of notation was only present for easy tasks [pie charts vs. decimals: *t*(28.53) = 9.48, *p* < 0.001] (i.e., not for dot patterns vs. fractions [*t*(32.28) = 0.39, *p* = 0.980]). In contrast, there were significant differences between easy and difficult tasks for both symbolic [fractions vs. decimals: *t*(29.71) = 18.08, *p* < 0.001] and non-symbolic [dot patterns vs. pie charts: *t*(28.70) = 8.65, *p* < 0.001] notations. This indicated that overall, easier tasks were compared faster than more difficult tasks (easy: *M* = 1.00 item/s, *SE* = 0.04 items/s vs. difficult: *M* = 0.54 item/s, *SE* = 0.04 items/s).

We also observed a significant interaction between notation and difficulty for ER, *χ*^*2*^(1) = 22.92, *p* < 0.001. It indicated that—although all pairwise comparisons were significant (dot patterns vs. fractions: *z* = 2.92, *p* = 0.019; dot patterns vs. pie charts: *z* = 8.33, *p* < 0.001; dot patterns vs. decimals: *z* = 8.09, *p* < 0.001; fractions vs. pie charts: *z* = 4.84, *p* < 0.001; fractions vs. decimals: *z* = 7.14, *p* < 0.001; pie charts vs. decimals: *z* = 4.95, *p* < 0.001)—the difference between symbolic and non-symbolic notation was smaller for difficult as compared to easy comparisons (difficult: log odds = 0.57, *SE* = 0.23 vs. easy: log odds = 2.53, *SE* = 0.51). For reasons of readability, the following descriptions of results also incorporate ERs in percent. Accordingly, error rates for decimals were lowest (log odds: *M* = − 5.06, *SE* = 0.53; 1%), followed by pie charts (log odds: *M* = − 2.53, *SE* = 0.25; 7%) and fractions (log odds: *M* = − 1.53, *SE* = 0.24; 18%) and highest for dot patterns (log odds: *M* = − 0.86, *SE* = 0.24; 30%). Again, main effects of notation, *χ*^2^(1) = 27.73, *p* < 0.001, as well as difficulty, *χ*^2^(1) = 46.32, *p* < 0.001, were significant. The main effect of notation indicated that comparing symbolic proportions (log odds: *M* = − 3.30, *SE* = 0.32; 4%) was less error prone than comparing non-symbolic proportions (log odds: *M* = − 1.29, *SE* = 0.22; 16%). Furthermore, participants’ error rates were lower in easier (log odds: *M* = − 3.80, *SE* = 0.32; 2%) than in more difficult tasks (log odds: *M* = − 1.19, *SE* = 0.21; 23%).

### Results for appraisal questionnaire

The ANOVA on *threat* (i.e., negative emotions) revealed main effects of both notation, *F*(1, 24) = 21.45, *p* < 0.001, and difficulty, *F*(1, 24) = 31.93, *p* < 0.001. The main effect of notation indicated that participants rated proportions presented in non-symbolic notations (i.e., dot patterns and pie charts) more negative (i.e., threatening, M = 7.86, SE = 0.55, 95% CI [6.74, 8.98]) than proportions presented symbolically (i.e., fractions and decimals, M = 6.00, SE = 0.55, 95% CI [4.88, 7.12]). The main effect of difficulty indicated that participants rated difficult proportions (i.e., fractions and dot patterns) as more negative (i.e., threatening, M = 8.66, SE = 0.59, 95% CI [7.46, 9.86]) than easy proportions (i.e., pie charts and decimals, M = 5.20, SE = 0.59, 95% CI [4.00, 6.40]). The interaction between notation and difficulty was not significant, *F*(1, 24) = 1.78, *p* = 0.60.

The ANOVA on *challenge* (i.e., positive emotions) revealed a main effect of difficulty, *F*(1, 24) = 16.43, *p* < 0.001. The main effect of difficulty indicated that participants rated easy proportions more positive (i.e., challenging, pie charts and decimals, M = 4.73, SE = 0.24, 95% CI [4.24, 5.23]) than difficult proportions (i.e., fractions and dot patterns, M = 4.20, SE = 0.23, 95% CI [3.72, 4.68]). There was neither a main effect of notation, *F*(1, 24) = 2.11, *p* = 0.16, nor an interaction between notation and difficulty, *F*(1, 24) < 1, *p* = 0.44.

### Imaging results

The *F*-contrast of the ANCOVA revealed no supra-threshold clusters for the interaction between difficulty (easy/difficult) and notation (symbolic/non-symbolic).

**Fig. 3:**
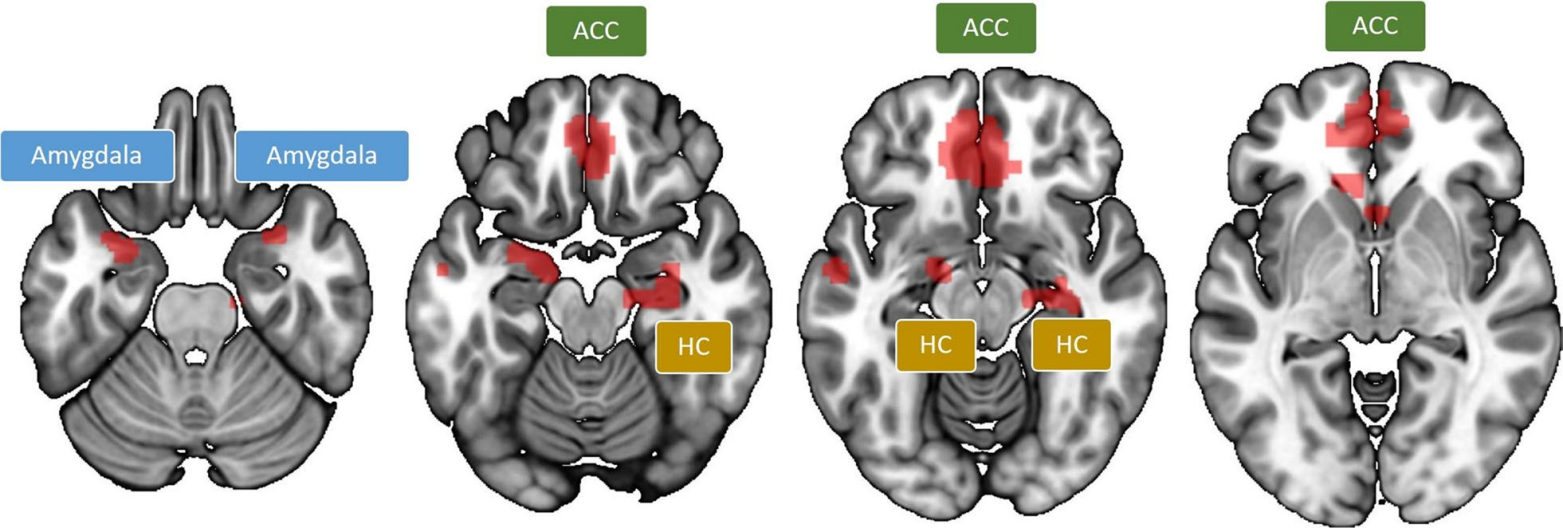
Negative emotional response to anticipating difficult math. Negative emotion network stronger associated with cues indicating upcoming difficult (including fractions and dots) than with cues indicating an easy proportion comparison task (involving pies and decimals). *ACC* anterior cingulate cortex, *HC* hippocampus

The analysis of the main effect of difficulty yielded the following results: Cues indicating a difficult upcoming task (dots, fractions) as compared to an easy task (pies, decimals) led to increased activation in a network including bilateral amygdala, bilateral ACC, bilateral hippocampus, left temporal gyrus and bilateral paracentral gyrus (Fig. 3, Table 1). For the opposite contrast (cues for easy vs. difficult tasks) no supra-threshold clusters were observed.

**Table 1:** Cortical regions more strongly activated in the conjunction of viewing at cues for upcoming dots and fractions (difficult conditions) compared to pies and decimals (easy conditions), controlled for math anxiety values as measured with the AMAS

Contrast	Brain region	MNI (x, y, z)			Cluster size	t
Difficult vs. easy	LH anterior cingulate cortex	− 5	43	− 15	446	5.59
	RH anterior cingulate cortex*	3	31	− 15		4.64
	LH amygdala	− 27	1	− 23	63	4.61
	RH amygdala	28	6	− 28	15	3.75
	RH hippocampus	31	− 10	− 18	66	4.13
	LH middle temporal gyrus	− 57	− 10	− 13	15	3.92
	LH paracentral gyrus	− 7	− 40	73	46	4.50
	RH postcentral gyrus	13	− 42	70	31	4.33

p_cluster-corr_ < 0.05 (k = 10 voxels)

*LH* left hemisphere; *MNI* Montreal Neurological Institute coordinates; *RH* right hemisphere; *: minor maximum; *t* t-value

The main effect of notation revealed no supra-threshold activation differences between conditions when corrected for multiple comparisons. However, as we had the hypothesis that symbolic vs. non-symbolic cues should activate the VNF, we also did the analysis at an uncorrected *p*-value of .001 and found activation in the left inferior temporal gyrus at MNI coordinates − 51, − 55, − 16 (*t *= 3.22).

Neither large nor small values of the covariate math anxiety (as reflected by participants’ AMAS scores) explained any suprathreshold cluster of activation. This means that math anxiety scores did not explain variance of the fMRI signal during cue presentation. For the sake of completeness, simple effects for each of the four cues (dots, fractions, pies, decimals) are given in Additional files 1, 2, 3, 4

## Discussion

The mere anticipation of doing difficult tasks is often associated with negative emotions (e.g., [5, 56]). However, in the case of math such a negative emotional response was proposed to be only observed in highly math-anxious participants according to Lyons and Beilock [8]. In the present study, we aimed at evaluating (i) that negative emotional responses in anticipation of a difficult math task is a general response, which can be seen in non-math-anxious individuals as well and (ii) that it is the regulation of the initial emotional response which is crucial for later task performance.

Subjective and objective behavioral measures confirmed that our manipulation of difficulty was successful. Additionally, difficult items were subjectively rated as more negative (i.e., threatening), while easy items were rated as more positive (i.e., challenging). More specifically, our non-math-anxious participants explicitly indicated that more difficult comparisons of fractions and dot patterns were associated with more negative feelings. This further supports the notion that activation associated with the anticipation of difficult trials indeed reflects processes related to negative emotions.

In line with our hypothesis, imaging data revealed a common (sub-)network associated with the processing of negative emotions activated independently of notation (symbolic/non-symbolic) and math anxiety scores, but dependent on the degree of difficulty. In our non-math-anxious participants, cues indicating a more difficult upcoming task led to activation in a network comprising bilateral amygdala and hippocampus, which has been previously associated with the processing of negative emotions (e.g., [57, 58]). In line with our hypothesis, these participants also revealed activation in areas associated with cognitive control such as anterior cingulate cortex (ACC). Networks of cognitive control have been suggested to be involved in emotion regulation during numerical tasks (e.g., [8, 9]). Importantly, when examining whether fMRI signal was predicted by the degree of math anxiety (as reflected by participants’ AMAS scores) in our non-math anxious sample, neither high nor low values of math anxiety explained any suprathreshold cluster of activation. This means that math anxiety scores did not explain fMRI signal during cue presentation. In other words, while the activation observed in the emotion processing network seems to be associated with the anticipation of an upcoming difficult (math) task, it was clearly not associated with participants’ degree of math anxiety.

Finally, in line with our expectations we only found specific activation of the VNF area following symbolic numerical cues, whereas IPS activation was not observed specifically for symbolic notation.

### Affective responses associated with cues for difficult numerical tasks

Behavioral data indicated that magnitude comparison performance indeed differed significantly as a function of task difficulty. Comparisons of fractions and dot patterns were responded to slower and with more errors than comparisons of decimals and pies. Imaging data revealed that, when presented with cues indicating a difficult upcoming task, our non-math-anxious participants showed activation in a network associated with the processing of negative emotions comprising hippocampus and amygdala, while math anxiety scores did not modulate the fMRI signal. Lyons and Beilock [8] recently reported activation of a similar network comprising hippocampus in anticipation of doing math for individuals with high math anxiety and claimed that networks of emotion processing should not be observed to be active for non-math-anxious individuals. However, it has been shown in both human lesion and neuroimaging studies that the amygdala, which we additionally found active, plays a crucial role in classical fear conditioning and fear-potentiated startle [57, 59]; for a review see [26]. In turn, amygdala and hippocampus were shown to work closely integrated in emotional responses (e.g., [58]). Therefore, it is likely that the network observed in the present study resembles a network for emotion processing. Involvement of networks associated with the processing of emotions and pain when anticipating doing difficult numerical tasks [9] was further substantiated by a conjunction analysis between cues indicating upcoming dot and fraction magnitude comparison tasks (see Additional files 5, 6).

It needs to be acknowledged, however, that we only measured non-math-anxious participants. As such we cannot be sure that the networks identified in non-math-anxious participants when they anticipated a difficult math task are identical to those recently reported for high math-anxious participants [8, 9]. However, math anxiety scores did not explain variance of the fMRI signal. Furthermore, the fact that we observed activation of a network typically associated with the processing of (negative) emotions suggests an emotional reaction of the present non-math-anxious participants anticipating the upcoming difficult math tasks. Additionally, it is important to note that these results do not reflect absolute activation of the network while participants anticipated the difficult math task but the relative stronger activation of these areas in anticipation of a difficult vs. an easier math task. Finally, the results on the neural level are substantiated by the results of the appraisal questionnaire that suggest that the respective emotions may have been negative indeed (see also [17] for physiological data).

Importantly, cues indicating difficult upcoming tasks also led to activation in bilateral anterior cingulate cortex. ACC is part of the cognitive control network, which is in turn suggested to play a key role in emotion regulation and the regulation of negative emotions via reappraisal (e.g., [24]); for a review see [25, 60]. This suggests that in our non-math-anxious participants the initial negative emotional response seemed to be sufficiently regulated so that the participants were not identified as math anxious in the Abbreviated Math Anxiety Scale [35].

This idea of a counter play between initial negative emotional responses and emotion regulation is in line with recent work on math anxiety (e.g., [8, 22]). The authors suggested that efficient regulation and control mechanisms of negative emotions before starting a task should increase math performance in highly math anxious participants. Therefore, Maloney and Beilock [22] suggested that training highly math anxious individuals in emotion regulation might limit negative effects of math anxiety on math performance. In turn, better emotion regulation may even lead to better math performance in highly math-anxious people. However, we suggest that negative emotions in anticipation of doing difficult math are to be expected in general, which does not only occur in highly math-anxious but also in non-math-anxious participants. This means that the (successful) regulation of an initial emotional response by means of cognitive control processes might be crucial for actual math performance. In case emotion regulation is sufficient, the task at hand can be performed with all available cognitive resources [21], so that performance does not have to be impaired even in highly math-anxious participants [8]. We suggest that successful emotion regulation accompanied by the experience of better performance may in turn reduce negative feelings such as fear in anticipation of doing math so that individuals with better cognitive control/emotion regulation should be less likely to develop math anxiety. However, we wish to note that in the current study we did not directly assess emotion regulation or demonstrate a relationship between emotion regulation and task performance. Therefore, this interpretation has to remain speculative; nevertheless, this interpretation is in line with the idea already proposed by Lyons and Beilock [8, 9] that it may not be the initial negative affective response, which is indicative of math anxiety, but the inability to regulate this response effectively, which in turn may lead to resource depletion and reduced math performance in those with high math anxiety.

### Responses associated with cues containing arabic digits

We observed no general effect of notation. However, only symbolic cues (i.e., fractions and decimals) led to significant number-specific activation in the posterior inferior temporal gyrus (pITG). This region was reported to selectively respond to visually presented numerals using intraoperative electrocorticography recordings [61] and fMRI [62]. Accordingly, the authors suggested that the visual number form might be represented in the bilateral inferior temporal gyri rather than the bilateral fusiform gyri as proposed by Dehaene and Cohen [36, 63]. Recently, Daitch et al. [64], substantiated this claim for a subregion within the pITG selectively responding to numerals compared to morphologically similar stimuli using electrocorticography. Therefore, the present data are in line with the notion that pITG might indeed be involved in the processing of visual numerals, while the fusiform gyrus may be less selectively involved in the detection and early non-semantic higher order visual analysis of symbolic and non-symbolic patterns.

Interestingly and in contrast to pITG, IPS activation observed in the current study was neither specific for symbolic notation nor for task difficulty. This is consistent with the findings of Shi et al. [65], who reported IPS activation associated with the mere anticipation of numerical magnitude comparison without the actual presentation of numbers themselves. As regards notation-related effects, our results are also in line with the literature because the IPS is generally assumed to subserve a notation-independent representation of number magnitude [30–33].

### Implications for the concept of math anxiety

Our results are fully consistent with the account proposed by Lyons and Beilock [8, 9] that more difficult numerical tasks may elicit negative emotions or even associations with pain, which require counter regulation by processes of cognitive control so that individuals can keep their focus on accomplishing the task at hand. However, the present results indicate that this may not be exclusively the case for math-anxious individuals, as suggested by Lyons and Beilock [8, 9]. Instead, these data imply that the account of initial (negative) emotional responses and the need for subsequent counter regulation of these responses generalizes also to the case of non-math-anxious participants. Thus, a neuronal signature of negative emotions in anticipation of doing difficult math seem to be a rather general response taking place in the human brain, even in individuals not diagnosed with math-anxiety. This would be in line with previous findings that individuals generally become more anxious when anticipating a relatively difficult task and thus require emotional regulation [56, 66].

However, we agree with Lyons and Beilock [8, 9] who question whether or not this initial negative response actually hinders math performance might depend on the ability to regulate these emotions. We suggest that successful regulation of negative emotions not only helps to solve the actual tasks (reflected by typical task performance), as proposed by Lyons and Beilock [8, 9], but that, depending on cognitive predisposition and other factors such as social influences [22], successful cognitive regulation might also prevent the development of math anxiety. This might have been the case for our non-math-anxious participants. Therefore, our study supports the idea that it should be more effective to train math-anxious individuals in emotion regulation to foster cognitive control processes (see for example [20]) than to train mathematical tasks themselves, as previously proposed by Lyons and Beilock ([8]; see also [22]).

### Limitations of the present study

It is important to note that there are some aspects that need to be considered when interpreting the results of the current study. First, we did not assess a non-mathematical control condition. Therefore, we cannot and do not want to make any claims on whether the effect observed is a general effect of difficulty or indeed specific to the anticipation of difficult math tasks. However, we would suggest that the idea of a negative emotional response in anticipation of doing difficult tasks might rather be a general mechanism instead of a mechanism specific to vulnerable individuals. Future studies are needed to decide whether this finding is limited to the case of mathematical tasks or not.

Furthermore, we evaluated non-math-anxious participants only. Therefore, we cannot tell for sure whether the observed activation patterns in anticipation of doing difficult (math) tasks are identical in low and highly math-anxious participants. However, we want to point out that the general neural response pattern we observed seems to indicate a (negative) emotional response in non-math-anxious participants as well as processes of emotion regulation. Therefore, we suggest that the overall response patterns seem to show at least similar reactions (negative emotions, emotion regulation) to the anticipation of difficult tasks as they have already been shown for highly math-anxious participants. As such, we agree with the account proposed by Lyons and Beilock [8] who described coupled processes of negative emotions with subsequent emotion regulation in anticipation of doing math. Nevertheless, we would like to suggest that this account may also generalize to the case of non-math-anxious individuals. Future studies would be desirable to evaluate this suggestion.

Finally, we wish to note that in the current study we did not directly assess emotion regulation or demonstrate a relationship between emotion regulation and task performance. Therefore, our interpretation that it is the ability to sufficiently regulate emotions that prevents the initial negative response to difficult math tasks from actually hindering math performance must remain speculative for the time being. Nevertheless, we wish to note that our interpretation is generally in line with ideas already proposed by Lyons and Beilock [8, 9].

## Conclusion

When anticipating a difficult upcoming task, non-math-anxious participants revealed activation within a network associated with the processing of negative emotions. However, whether or not this initial negative response actually hinders math performance seems to depend on the ability to sufficiently regulate these emotions. While the relevance of such emotion regulation for typical task performance has been suggested before for the case of highly math-anxious individuals, we propose to extend this account to the case of non-math-anxious individuals. We suggest that the observed pattern of neuronal responses on emotion processing and emotion regulation mechanisms seem to indicate a general mechanism rather than a mechanism specific to math-anxious individuals. As such successful emotion regulation might be a general prerequisite for cognitive performance when facing demanding numerical tasks.

## Additional files

**Additional file 1: Fig S1.** Preparation network associated with cues indicating an upcoming magnitude comparison task with either dots or fractions. The color bar indicates *t*-values (*p*_cluster-corr_ < .05, cluster size *k* = 10). A negative emotion network can be observed including amygdala, hippocampus, insula, and ACC as well as the preparation network (fusiform gyrus, IPS).

**Additional file 2: Table S1.** Cortical regions more strongly activated when looking at cues indicating an upcoming dot or fraction magnitude comparison task compared to rest. *p*_cluster-corr_ < .05 (*k* = 10 voxels); LH: left hemisphere; MNI: Montreal Neurological Institute coordinates; RH: right hemisphere; *t* = *t*-value. *Minor maximum.

**Additional file 3: Fig S2.** Preparation network for cues indicating an upcoming magnitude comparison task with either pies or decimals. The color bar indicates *t*-values (*p*_cluster-corr_ < .05, cluster size *k* = 10).

**Additional file 4: Table S2.** Cortical regions more strongly activated when viewing at cues indicating an upcoming pie or decimal magnitude comparison task compared to rest. *p*_cluster-corr_ < .05 (*k* = 10 voxels); LH: left hemisphere; MNI: Montreal Neurological Institute coordinates; RH: right hemisphere; *t* = *t*-value. *Minor maximum.

**Additional file 5: Fig S3.** Conjunction of cues indicating a difficult (involving dots and fractions) or easy (involving pies and decimals) upcoming magnitude comparison task. The color bar indicates *t*-values (*p*_cluster-corr_ < .05, cluster size *k* = 10).

**Additional file 6: Table S3.** Cortical regions more strongly activated in the conjunction of viewing at cues for upcoming dots and fractions compared to rest. *p*_cluster-corr_ < .05 (*k* = 10 voxels); LH: left hemisphere; MNI: Montreal Neurological Institute coordinates; RH: right hemisphere; *t* = *t*-value.

## Abbreviations

DLPFC: dorsolateral prefrontal cortex
ACC: anterior cingulate cortex
IPS: intraparietal sulcus
HC: hippocampus
pITG: posterior inferior temporal gyrus
VNF: visual number form
AMAS: Abbreviated Math Anxiety Scale
