# Supplementary figures and images for: Anticipation of difficult tasks: neural correlates of negative emotions and emotion regulation
Source: Behav Brain Funct. 2019 Mar 18;15:4. doi: 10.1186/s12993-019-0155-1 (PMC6421679; doi:10.1186/s12993-019-0155-1)

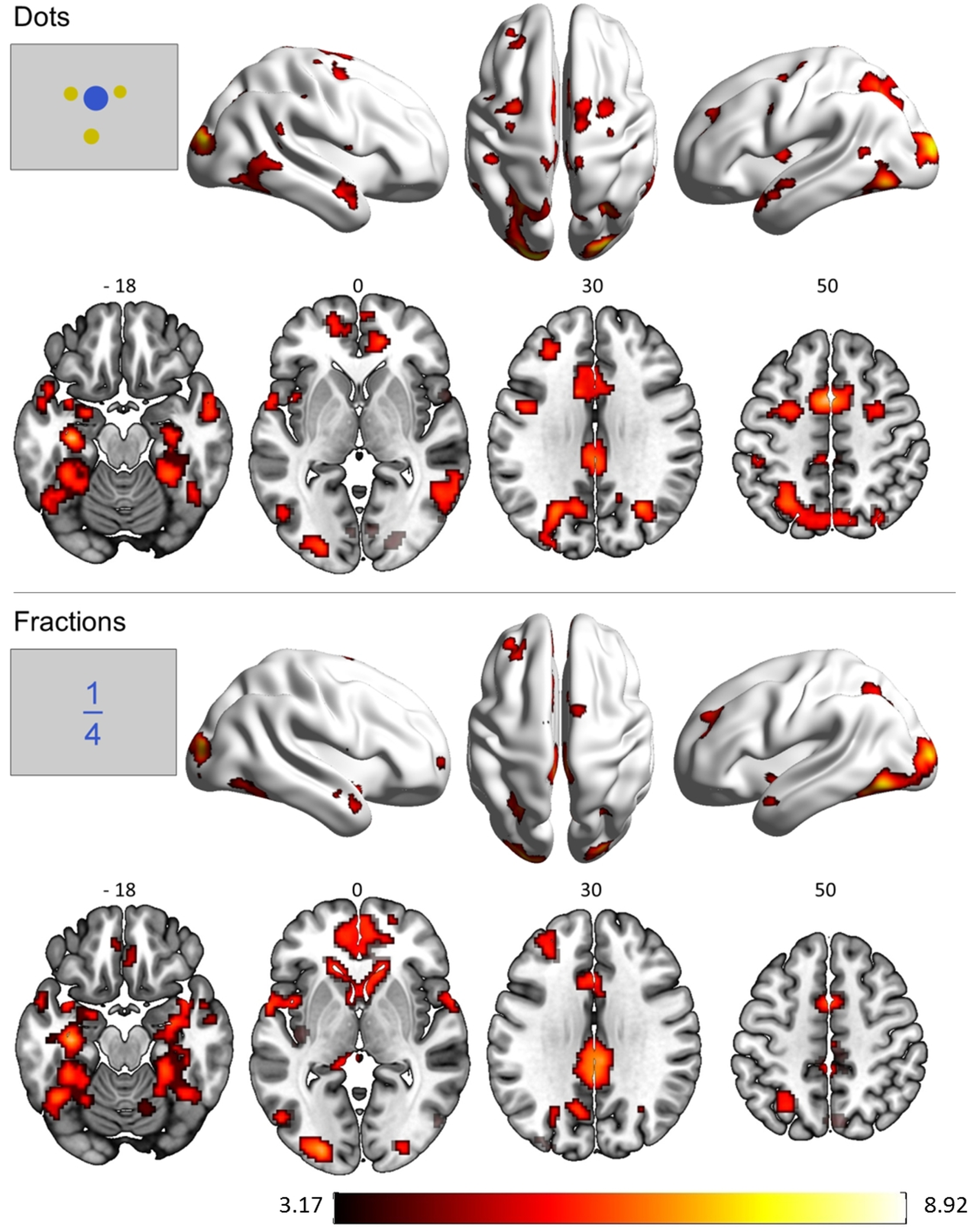

Supplement: Supplementary file 1 — Additional file 1: Fig S1. Preparation network associated with cues indicating an upcoming magnitude comparison task with either dots or fractions. The color bar indicates t-values (pcluster-corr < .05, cluster size k = 10). A negative emotion network can be observed including amygdala, hippocampus, insula, and ACC as well as the preparation network (fusiform gyrus, IPS). [file 12993_2019_155_MOESM1_ESM.tif]

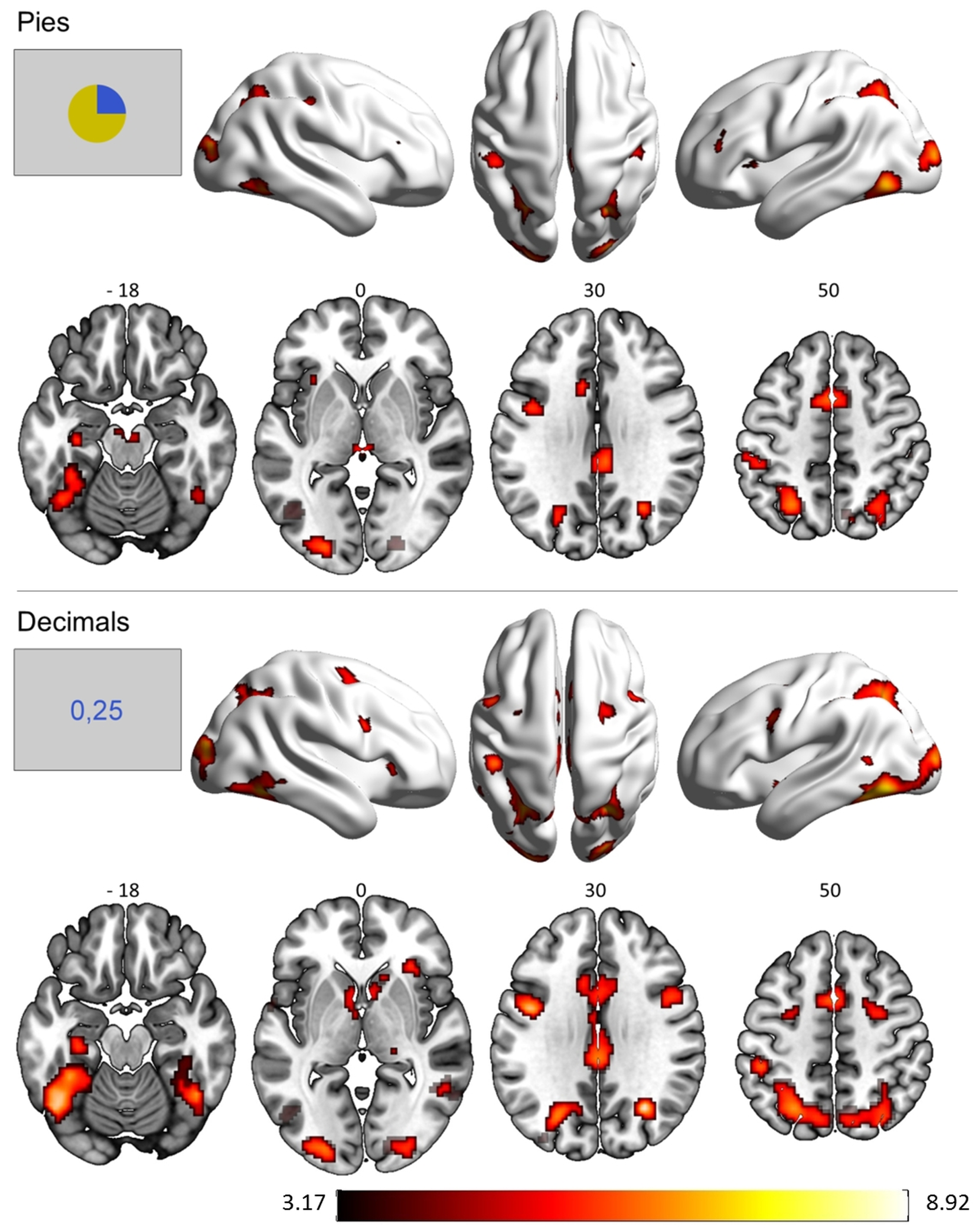

Supplement: Supplementary file 3 — Additional file 3: Fig S2. Preparation network for cues indicating an upcoming magnitude comparison task with either pies or decimals. The color bar indicates t-values (pcluster-corr < .05, cluster size k = 10). [file 12993_2019_155_MOESM3_ESM.tif]

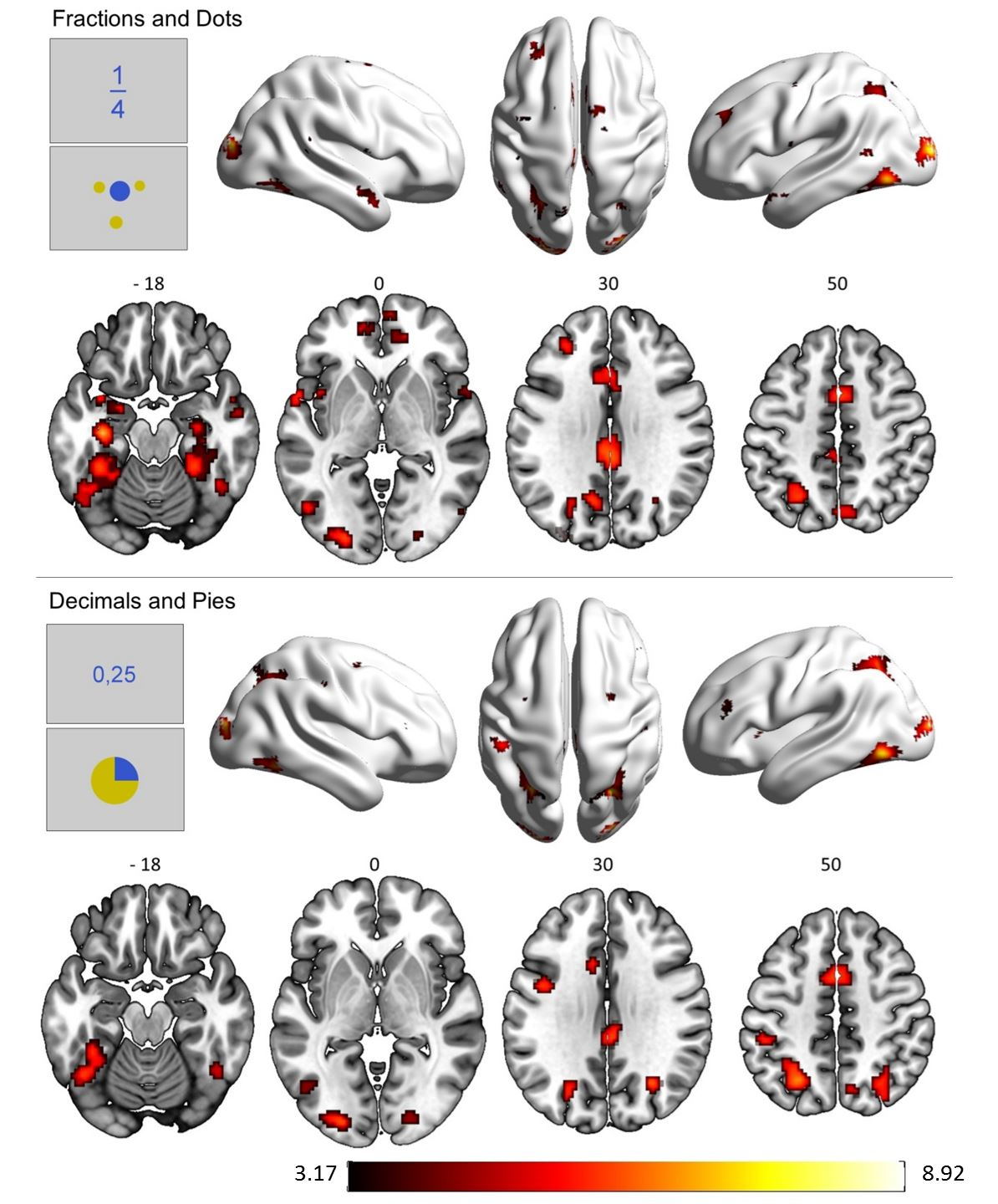

Supplement: Supplementary file 5 — Additional file 5: Fig S3. Conjunction of cues indicating a difficult (involving dots and fractions) or easy (involving pies and decimals) upcoming magnitude comparison task. The color bar indicates t-values (pcluster-corr < .05, cluster size k = 10). [file 12993_2019_155_MOESM5_ESM.tif]
