# Supplementary material for: Anticipation of difficult tasks: neural correlates of negative emotions and emotion regulation
Source: Behav Brain Funct. 2019 Mar 18;15:4. doi: 10.1186/s12993-019-0155-1 (PMC6421679; doi:10.1186/s12993-019-0155-1)
Supplement: Supplementary file 2 — Additional file 2: Table S1. Cortical regions more strongly activated when looking at cues indicating an upcoming dot or fraction magnitude comparison task compared to rest. pcluster-corr < .05 (k = 10 voxels); LH: left hemisphere; MNI: Montreal Neurological Institute coordinates; RH: right hemisphere; t = t-value. *Minor maximum. [file 12993_2019_155_MOESM2_ESM.docx]

**Table S1**

|  |  |  |  |  |  |  |
| --- | --- | --- | --- | --- | --- | --- |
|  |  |  |  |  |  |  |
| Contrast | Brain region | MNI (x, y, z) | | | Cluster size | *t* |
|  |  |  |  |  |  |  |
|  |  |  |  |  |  |  |
| Cue dots vs. baseline | LH amygdala | -30 | -2 | -30 | 24 | 4.47 |
|  | LH insula | -40 | -2 | 8 | 46 | 4.25 |
|  | LH hippocampus | -35 | -20 | -18 | 48 | 6.45 |
|  | RH hippocampus | 33 | -17 | -20 | 18 | 5.00 |
|  | RH anterior cingulate cortex | 13 | 43 | 0 | 13 | 4.68 |
|  | LH intraparietal sulcus (hIP3) | -25 | -65 | 43 | 311 | 5.48 |
|  | LH supplementary motor area | -2 | 6 | 53 | 559 | 6.71 |
|  | RH supplementary motor area* | 11 | 6 | 45 |  | 5.73 |
|  | LH middle frontal gyrus | -27 | -2 | 53 | 27 | 4.66 |
|  | LH middle frontal gyrus | -30 | 36 | 30 | 20 | 4.25 |
|  | RH middle temporal gyrus | 66 | -47 | 5 | 105 | 4.84 |
|  | RH middle temporal gyrus | 58 | -2 | -15 | 27 | 4.48 |
|  | LH temporal pole | -47 | 11 | -23 | 43 | 4.51 |
|  | LH middle temporal gyrus* | -45 | 3 | -25 |  | 4.47 |
|  | RH fusiform gyrus | 41 | -42 | -28 | 291 | 7.00 |
|  | LH fusiform gyrus | -37 | -45 | -18 | 58 | 4.64 |
|  | LH retrosplenial cortex | 1 | -35 | 30 | 103 | 4.88 |
|  | RH precuneus | 8 | -70 | 40 | 54 | 4.54 |
|  | LH inferior occipital gyrus | -47 | -70 | -13 | 110 | 6.24 |
|  | RH superior occipital gyrus | 31 | -70 | 40 | 110 | 5.59 |
|  | LH middle occipital gyrus | -20 | -95 | 10 | 304 | 8.92 |
|  | RH middle occipital gyrus | 28 | -90 | 15 | 165 | 7.09 |
|  |  |  |  |  |  |  |
| Cue fractions vs. | LH amygdala | -22 | -5 | -23 | 31 | 5.23 |
| baseline | LH insula | -40 | -15 | 3 | 11 | 4.42 |
|  | RH insula | 41 | 6 | -10 | 10 | 4.24 |
|  | LH hippocampus | -35 | -20 | -18 | 85 | 6.32 |
|  | RH hippocampus | 31 | -10 | -18 | 15 | 4.64 |
|  | RH anterior cingulate cortex | 3 | 40 | -5 | 34 | 5.07 |
|  | LH intraparietal sulcus (hIP3) | -27 | -63 | 40 | 77 | 5.09 |
|  | RH supplementary motor area | 1 | 3 | 55 | 287 | 5.45 |
|  | LH supplementary motor area* | -7 | 13 | 45 |  | 4.86 |
|  | RH caudate nucleus | 8 | 18 | 5 | 535 | 5.49 |
|  | LH middle frontal gyrus | -30 | 43 | 35 | 29 | 4.64 |
|  | LH middle frontal gyrus | -35 | 48 | 23 | 17 | 4.59 |
|  | LH superior temporal gyrus | -62 | -22 | 13 | 23 | 4.64 |
|  | LH superior temporal gyrus | -60 | 3 | 0 | 46 | 4.52 |
|  | RH medial temporal pole | 36 | 11 | -33 | 18 | 5.21 |
|  | LH post. inferior temporal gyrus | -45 | -65 | -10 | 260 | 6.61 |
|  | RH post. inferior temporal gyrus | 51 | -67 | -10 | 17 | 4.22 |
|  | RH inferior temporal gyrus | 43 | -55 | -13 | 17 | 4.20 |
|  | RH fusiform gyrus | 33 | -40 | -25 | 191 | 5.27 |
|  | LH fusiform gyrus | -24 | -82 | -10 | 53 | 5.78 |
|  | LH precuneus | -12 | -65 | 33 | 92 | 4.67 |
|  | LH retrosplenial cortex | -2 | -40 | 25 | 274 | 5.92 |
|  | LH middle occipital gyrus | -22 | -95 | 8 | 255 | 7.65 |
|  | RH middle occipital gyrus | 28 | -90 | 13 | 129 | 6.78 |
|  |  |  |  |  |  |  |
