# Supplementary material for: Anticipation of difficult tasks: neural correlates of negative emotions and emotion regulation
Source: Behav Brain Funct. 2019 Mar 18;15:4. doi: 10.1186/s12993-019-0155-1 (PMC6421679; doi:10.1186/s12993-019-0155-1)
Supplement: Supplementary file 4 — Additional file 4: Table S2. Cortical regions more strongly activated when viewing at cues indicating an upcoming pie or decimal magnitude comparison task compared to rest. pcluster-corr < .05 (k = 10 voxels); LH: left hemisphere; MNI: Montreal Neurological Institute coordinates; RH: right hemisphere; t = t-value. *Minor maximum. [file 12993_2019_155_MOESM4_ESM.docx]

**Table S2**

|  |  |  |  |  |  |  |
| --- | --- | --- | --- | --- | --- | --- |
|  |  |  |  |  |  |  |
| Contrast | Brain region | MNI (x, y, z) | | | Cluster size | *t* |
|  |  |  |  |  |  |  |
|  |  |  |  |  |  |  |
| Cue pies vs. baseline | RH intraparietal sulcus (hIP3) | 30 | -56 | 45 | 246 | 6.46 |
|  | LH intraparietal sulcus (hIP2) | -48 | -42 | 50 | 58 | 4.77 |
|  | LH superior parietal lobe (PSPL) | -22 | -72 | 55 | 321 | 5.88 |
|  | LH supplementary motor area | -5 | 6 | 55 | 94 | 5.28 |
|  | LH middle cingulate cortex | -10 | 13 | 38 | 290 | 4.57 |
|  | LH inferior temporal gyrus | -47 | -67 | -10 | 158 | 6.33 |
|  | RH inferior temporal gyrus | 46 | -55 | -10 | 94 | 5.68 |
|  | RH fusiform gyrus | 33 | -37 | -25 | 109 | 5.14 |
|  | LH fusiform gyrus | -37 | -45 | -18 | 344 | 4.22 |
|  | RH retrosplenial cortex | 6 | -35 | 30 | 34 | 4.89 |
|  | LH middle occipital gyrus | -20 | -95 | 10 | 377 | 7.37 |
|  | RH middle occipital gyrus | 28 | -90 | 15 | 133 | 6.36 |
|  |  |  |  |  |  |  |
| Cues decimals vs. | LH hippocampus | -32 | -25 | -18 | 10 | 4.43 |
| baseline | LH anterior cingulate cortex | 1 | 11 | 30 | 17 | 4.14 |
|  | LH middle cingulate cortex | -10 | 13 | 38 | 33 | 5.23 |
|  | LH intraparietal sulcus (hIP3) | -25 | -62 | 48 | 471 | 6.96 |
|  | LH intraparietal sulcus (hIP2) | -45 | -39 | 43 | 66 | 5.48 |
|  | RH intraparietal sulcus (hIP3) | 33 | -52 | 43 | 494 | 4.29 |
|  | LH supplementary motor area | -2 | 6 | 53 | 67 | 4.91 |
|  | LH inferior frontal gyrus (44) | -45 | 1 | 30 | 97 | 6.19 |
|  | RH inferior frontal gyrus (45) | 46 | 8 | 28 | 22 | 4.61 |
|  | RH middle frontal gyrus | 31 | -2 | 53 | 14 | 4.32 |
|  | LH post. inferior temporal gyrus | -47 | -55 | -15 | 322 | 6.56 |
|  | RH post. inferior temporal gyrus | 46 | -57 | -13 | 291 | 6.41 |
|  | RH retrosplenial cortex | 1 | -34 | 28 | 93 | 5.25 |
|  | LH fusiform gyrus | -17 | -90 | -10 | 326 | 8.83 |
|  | RH fusiform gyrus | 18 | -90 | -8 | 276 | 7.23 |
|  | LH middle occipital gyrus | -20 | -95 | 8 | 1164 | 7.29 |
|  | LH inferior occipital gyrus | -47 | -65 | -15 | 446 | 7.32 |
|  | RH middle occipital gyrus | 28 | -90 | 13 | 408 | 7.23 |
|  |  |  |  |  |  |  |
