# Supplementary material for: Anticipation of difficult tasks: neural correlates of negative emotions and emotion regulation
Source: Behav Brain Funct. 2019 Mar 18;15:4. doi: 10.1186/s12993-019-0155-1 (PMC6421679; doi:10.1186/s12993-019-0155-1)
Supplement: Supplementary file 6 — Additional file 6: Table S3. Cortical regions more strongly activated in the conjunction of viewing at cues for upcoming dots and fractions compared to rest. pcluster-corr < .05 (k = 10 voxels); LH: left hemisphere; MNI: Montreal Neurological Institute coordinates; RH: right hemisphere; t = t-value. [file 12993_2019_155_MOESM6_ESM.docx]

**Table S3**

|  |  |  |  |  |  |  |
| --- | --- | --- | --- | --- | --- | --- |
| Contrast | Brain region | MNI (x, y, z) | | | Cluster size | *t* |
|  |  |  |  |  |  |  |
|  |  |  |  |  |  |  |
| Conjunction | LH amygdala | -30 | -2 | -30 | 84 | 5.40 |
| cues dots and fractions | LH hippocampus | -35 | -20 | -18 | 195 | 6.32 |
|  | LH insula | -42 | 8 | 0 | 16 | 3.57 |
|  | RH insula | 63 | 3 | 10 | 10 | 3.55 |
|  | RH anterior cingulate cortex | 13 | 46 | -3 | 14 | 4.20 |
|  | RH middle cingulate cortex | 3 | 8 | 43 | 408 | 5.45 |
|  | LH intraparietal sulcus (hIP3) | -27 | -65 | 40 | 429 | 5.09 |
|  | RH intraparietal sulcus (hIP3) | 31 | -60 | 45 | 31 | 3.90 |
|  | LH fusiform gyrus | -37 | -42 | -20 | 367 | 6.24 |
|  | RH fusiform gyrus | 31 | -42 | -23 | 396 | 5.09 |
|  | RH lingual gyrus | 21 | -90 | -8 | 77 | 4.87 |
|  | LH lingual gyrus | 8 | -82 | -10 | 15 | 3.85 |
|  | RH retrosplenial cortex | 1 | -35 | 30 | 206 | 4.88 |
|  | RH supplementary motor area | 13 | 1 | 68 | 104 | 3.64 |
|  | LH middle frontal gyrus | -30 | 41 | 33 | 64 | 4.18 |
|  | LH middle frontal gyrus | -32 | 48 | 23 | 15 | 3.91 |
|  | LH temporal pole | -55 | 6 | -5 | 74 | 4.04 |
|  | RH temporal pole | 58 | 8 | -5 | 10 | 3.57 |
|  | RH medial temporal pole | 38 | 8 | -33 | 22 | 4.13 |
|  | LH superior temporal gyrus | -62 | -25 | 13 | 23 | 3.84 |
|  | RH middle temporal gyrus | 51 | 6 | -25 | 19 | 3.74 |
|  | RH middle temporal gyrus | 56 | -5 | -18 | 10 | 3.44 |
|  | LH superior medial gyrus | -15 | 51 | 0 | 11 | 3.44 |
|  | RH middle orbital gyrus | 8 | 61 | -3 | 11 | 3.49 |
|  | LH middle occipital gyrus | -22 | -95 | 8 | 412 | 7.59 |
|  | RH middle occipital gyrus | 28 | -90 | 13 | 163 | 6.63 |
|  |  |  |  |  |  |  |
| Conjunction | RH intraparietal sulcus (hIP3) | 30 | -65 | 42 | 221 | 6.46 |
| cues pies and decimals | LH intraparietal sulcus (hIP3) | -25 | -65 | 40 | 313 | 5.88 |
|  | LH intraparietal sulcus (hIP2) | -45 | -35 | 43 | 98 | 4.75 |
|  | LH fusiform gyrus | -37 | -45 | -18 | 325 | 6.33 |
|  | RH fusiform gyrus | 47 | -58 | -10 | 175 | 5.68 |
|  | RH fusiform gyrus | 33 | -37 | -25 | 55 | 3.86 |
|  | RH lingual gyrus | 21 | -90 | -8 | 95 | 5.14 |
|  | LH supplementary motor area | -2 | 6 | 53 | 189 | 4.91 |
|  | LH inferior frontal gyrus (44) | -42 | 1 | 28 | 35 | 4.38 |
|  | RH retrosplenial cortex | 3 | -35 | 30 | 67 | 4.42 |
|  | RH precuneus | 11 | -67 | 43 | 34 | 3.83 |
|  | LH middle occipital gyrus | -20 | -95 | 8 | 323 | 7.11 |
|  | RH middle occipital gyrus | 28 | -90 | 15 | 121 | 6.36 |
|  |  |  |  |  |  |  |
|  |  |  |  |  |  |  |
